# Oral Health Care of Older Adults in Hong Kong

**DOI:** 10.3390/geriatrics6040097

**Published:** 2021-10-08

**Authors:** Alice Kit Ying Chan, Manisha Tamrakar, Katherine Chiu Man Leung, Chloe Meng Jiang, Edward Chin Man Lo, Chun-Hung Chu

**Affiliations:** Faculty of Dentistry, The University of Hong Kong, Hong Kong, China; dralice@hku.hk (A.K.Y.C.); manee626@hku.hk (M.T.); kcmleung@hku.hk (K.C.M.L.); cmjiang@hku.hk (C.M.J.); hrdplcm@hku.hk (E.C.M.L.)

**Keywords:** older adult, elderly, oral health, prevention, silver diamine fluoride, caries

## Abstract

The older adult population is increasing both in number and in proportion worldwide. In Hong Kong, the number of people aged 65 or above is expected to reach 2.5 million in 2039, thus becoming one-third of the population. With this growing population, the need for dental care among older adults is expected to surge. Oral health care is one of the government’s core policy agendas and the Department of Health has emphasised its importance. It has implemented a number of policies, such as increasing the number of dental training places, setting up an expert group for oral health care policy planning, and conducting regular oral health surveys of the population. It is subsidizing several programmes, including the Elderly Health Care Voucher Scheme, Community Care Fund Elderly Dental Assistance Programme, Outreach Dental Care Programme, and Comprehensive Social Security Assistance Programme, in order to promote oral health care in older adults. These programmes have received support and positive feedback from both the public and dental service providers. The purpose of this review is to provide an overview of the oral health care of older adults in Hong Kong and recommendations to enhance their effectiveness.

## 1. Introduction

Global aging is a major public health issue, and Hong Kong is no exception [1,2]. The older adult population in Hong Kong has grown rapidly in number and proportion. In fact, the number of older adults (aged 65 or above) is expected to double from 1.2 million in 2019 to 2.5 million in 2039, thus becoming one-third of the population [2]. This trajectory can be explained by an increase in life expectancy, as well as a decrease in the fertility rate. Life expectancy in Hong Kong has been steadily rising over the past half century to 85.3, which is the longest in the world [3]. Moreover, the fertility rate has dropped during the past few decades and reached its lowest number, 868 per 1000 women, in 2020 [4,5]. This changes the age structure in Hong Kong’s population, which is now characterized by a shrinking young population and an expanding older adult population [6].

During aging, people inevitably experience functional, cognitive, and physical impairments, due to physiological changes, multimorbidity, and polypharmacy [7]. The impact on general health increases the burden in an already overloaded health care system and society [8] as oral and general health are interrelated [9]. In light of this, preserving a functional dentition may be associated with a better quality of life, delayed frailty, dependence, and mortality [9,10]. Due to the surging demand for older adult health care, the Hong Kong government has implemented several policies for addressing both general health and oral health issues in the older adult population [11,12]. This review aims to provide an overview of the oral health care of older adults (aged 65 or above) in Hong Kong and to provide recommendations to enhance their effectiveness.

## 2. Characteristics of Older Adults in Hong Kong

Social determinants are associated with the individuals’ oral health [13]. Multimorbidity, polypharmacy, cognitive impairment, and function impairment make older adults more vulnerable to oral diseases [7]. To institute better policy planning, the government conducts regular territory-wide surveys to understand the socio-demographics and characteristics of older adults (aged 65 or above) in Hong Kong.

### 2.1. Residence

According to the 2016 Population Bi-census [14], the majority of older adults (91.9%) lived in domestic households, whereas 8.1% lived in residential homes, hospitals, or penal institutions, etc. Among those in domestic households, 34.8% lived alone or with their spouse. However, having foreign domestic helpers taking care of older adults is a special phenomenon in Hong Kong. Thus, the Bi-census found that 13% of domestic households with older adults only hired foreign domestic helpers as caregivers. Given the above, most of our older adults are community dwellers, with one-third choosing not to live with any caregivers.

### 2.2. Medical Conditions

Based on the thematic household survey in 2019 [15], three-fourths (78%) of older adults had chronic health conditions, hypertension (52%) being the most common condition, followed by high cholesterol (26%), diabetes mellitus (23%) and heart diseases (9%). Most of them were hence under medication.

### 2.3. Cognitive Condition

As people age, changes occur in all parts of the body, including the brain, which leads to changes in the cognitive function [16]. A 2009 survey showed that 11% of community dwellers and 40% of institutionalized older adults in Hong Kong had difficulty with recalling what had happened five minutes ago [17]. Another study stated that 9% of community-dwelling older adults in Hong Kong suffered from mild cognitive impairment, whereas 9% suffered from mild dementia [18]. Cognitive impairment definitively affects their self-care ability and hence their oral health.

### 2.4. Financial Situation

According to the 2016 Population Bi-census, three-fourths of older adults were retired. The reported median monthly domestic income of households with older adults only was HK 6020 (USD 774; USD 1 = HK 7.8), which is similar to the fee charged for a single removable partial denture in a private dental clinic subsidized under the Community Care Fund for eligible older adults [14]. Older adults in Hong Kong had a high poverty rate of 30%, which was among the highest in the developed economies in 2014 [19]. In addition, the majority (81%) had no medical benefits provided by their employers or individually purchased medical insurance [15]. This greatly reduced regular dental attendance [15].

## 3. Oral Health Status of Older Adults in Hong Kong

The oral health of older adults in Hong Kong is far from satisfactory, with high prevalence in both caries and periodontal disease. According to the 2011 oral health survey [20], 48% of the non-institutionalized older adults (aged 65–74) had untreated dental caries, with 1.3 decayed teeth found per older adult. Almost 90% had bleeding gums and periodontal attachment loss of more than 4mm on at least half of their teeth. When compared with the 2001 survey, non-institutionalized older adults retained more teeth than they had a decade before, with 60% having more than 20 teeth (functional dentition), and the proportion of edentulous older adults reduced. This implies that people today tend to retain more teeth as they age, compared with years past; however, oral diseases are still common.

The older adult population’ oral health condition was worse in those receiving long-term care. Based on the 2011 oral health survey [20], more than half had untreated dental caries, with 2.2–3.0 decayed teeth per older adult. Almost all presented with periodontal problems, gum bleeding and an attachment loss of more than 4mm. When compared with the 2001 survey, the 2011 survey reported a lower proportion of institutionalized older adults with more than 20 teeth (functional dentition), as well as a higher proportion of institutionalized older adults with edentulism. This may be due to the higher proportion of older adults (aged 85 or above) in the 2011 survey. These data show that extraction was the common treatment option for managing dental problems in older adults under long-term care. In light of this, many may have chewing difficulties.

## 4. Dental Services for Older Adults in Hong Kong

In Hong Kong, the public health care system provides comprehensive medical services at a relatively low fee for the public, but the dental services are limited. People mainly receive dental services in the private sector and through the non-governmental charitable or religious organizations (NGO) [19]. In 2019, among individuals who had undergone dental consultations during the past 12 months, 71% received them in private dental clinics, whereas 24% received them in government dental clinics [15]. Most dental treatment costs were self-paid.

### 4.1. Public Sector

The public sector provides free dental services to civil servants/pensioners and their dependents. The general public receives limited emergency services in the government dental clinics or in the oral and maxillofacial surgery and dental units in public hospitals. Eleven government dental clinics in Hong Kong run general public sessions that provide free emergency dental services, which comprise of treating acute dental diseases, pain relief, and extractions (one tooth per visit) [21]. A quota system of 32-84 patients per session is in place, and hence, patients need to queue up fairly early for services [21]. Older adults (aged 61 or above) made up more than half (57%) of the service recipients in 2017-2018 [21]. If older adults are civil servants/pensioners, they and their spouses are eligible for dental services in the government dental clinics or the public hospitals that the Department of Health or Hospital Authority provide [22]. The dental services are free and comprehensive, including scaling, filling, and root canal treatment. Patients need to pay a nominal fee and material costs for prostheses, such as bridges, removable partial dentures, and full dentures [22].

Older adults who have special needs, have dental emergencies, or who are public hospital inpatients can receive specialist care and treatment from the oral and maxillofacial surgery and dental units in seven public hospitals [23]. These units manage complicated extractions or dentoalveolar surgery, oral pathology and medicine, maxillofacial trauma, benign or malignant oral tumours, etc. [24]. Patients are required to pay a nominal fee [25].

### 4.2. Private Sector

The private sector is the mainstay of dental service for the general public in Hong Kong. Irrespective of the type of practice, dental services in the private sector are often provided on a fee-for-service basis, and are delivered in private dental clinics, health management organizations and NGOs. Private dental clinics can take the form of solo practice with only one dentist in the clinic or group practice where several dentists work together as associates or in partnership. No regulations exist regarding dental treatment fees in Hong Kong exist; thus, there is a huge variation in dental charges and in the dental services provided [26]. Fees may vary by location, type of practice and dentist qualifications and specialties [27]. A health management organization is a larger-scale commercial group practice that mainly provides dental benefits for employees (and their dependents) of companies with whom they are under contract with them. Some companies may extend dental benefits to their long-serving retired employees under these dental schemes. Eligible employees and their dependents pay a premium to cover dental check-ups and treatments, such as scaling, simple restorations, and extractions. However, they need to pay out of pocket for more advanced dental treatment or specialist care. The dental clinics the NGOs operate deliver a similar scope of dental services to private practices but at lower fees; thus, targeting lower-socioeconomic groups or disadvantaged groups, such as older adults or those with physical handicaps [28].

## 5. Oral Health Care Policies for Older Adults

Providing comprehensive dental services requires substantial resources. Thus, the Hong Kong government places emphasis on oral health education and promotion and prioritizes resources to provide emergency dental services, as well as oral health services, for people with special dental care needs, such as older adults with physical or financial difficulties [21]. Income level affects an individual’s dental attendance [15]. Older adults are often retired or have low incomes, which restricts their dental access. Hence, the government has introduced a number of health care policies, aimed at improving older adults’ dental access and oral health. In addition to the existing Comprehensive Social Security Assistance Scheme, the government has implemented several other subsidized schemes, such as the Elderly Health Care Voucher Scheme, the Community Care Fund Elderly Dental Assistance Programme, and the Outreach Dental Care Programme, in order to provide dental services for older adults. Table 1 summarizes the government subsidized dental services schemes.

### 5.1. Comprehensive Social Security Assistance Scheme

In 1950, Social Welfare Department set up and funded the Comprehensive Social Security Assistance Scheme, in order to financially help disadvantaged people to meet their basic needs [29]. The scheme provides dental grants for its recipients who are aged 60 or above, disabled, or medically certified of ill health [21]. The scheme covers the actual dental treatment expenses up to an amount, which is currently around HK 13,000 (USD 1666) [19]. The recipient must first receive a quote from a designated dental clinic and then obtain an approval of payment from the Social Welfare Department prior to commencing dental treatment [29]. They need to pay on their own for the dental costs that exceed the covered amount on their own. A total of 8617 dental claims were approved in 2015–2016, with an average claim of HK 6222 (USD 798) per patient [19].

### 5.2. Elderly Health Care Voucher Scheme

The Elderly Health Care Voucher Scheme was introduced in 2009, with an aim to encourage older adults to seek primary health care in private sectors and supplement the existing overloaded public health system [30]. It started as a pilot scheme for older adults (aged 70 or above) and offered a HK 250 (USD 32) voucher at an annual basis. Due to the positive feedback it received, the government increased the annual voucher amount several times and lowered the scheme’s eligibility age [30]. From 2020, older adults (aged 65 or above) hold a valid Hong Kong Identity Card can receive an annual voucher of HK 2000 (USD 256), and the unused vouchers have no expiration date and can be accumulated up to HK 8000 (USD 1024) [30]. The vouchers can be spent on 10 types of primary health care services, including dental treatments. A total of 1219 dentists have registered in the scheme; in 2020, HK 277 million (USD 29.1 M) was claimed on dental services [31]. The Elderly Health Care Voucher Scheme is the only scheme that covers all older adults (aged 65 or above), regardless of background, financial status, and health condition.

### 5.3. Community Care Fund Elderly Dental Assistance Programme

The Community Care Fund Elderly Dental Assistance Programme was launched in 2012 with a goal to improve chewing efficiency of older adults who have financial difficulties, through providing removable dentures to replace missing teeth and related dental treatments [32]. It initially targeted at people aged 60 or above who were neither Comprehensive Social Security Assistance Scheme recipients, nor users of the home care services or home help service schemes the Social Welfare Department provides [32]. The programme was later expanded to include older adults who were recipients of Old Age Living Allowance (aged 65 or above) [33]. Eligible older adults are allowed to use the service only once. The scope of subsidized services includes oral examinations, scaling, restorations, extractions, and removable dentures. The service provided is free to older adults, and dentists are remunerated according to a predetermined fee schedule, with the maximum subsidy being HK 15,350 (USD 1968) per older adult in 2019 [34,35]. An evaluation report revealed the Community Care Fund Elderly Dental Assistance Programme was well received. In January 2020, 564 private dentists and 69 NGO-operated dental clinics were enlisted in the programme, and 71,440 older adults had participated in the programme [35]. The programme further expanded in July 2021 to include more dental service items, such as root canal therapy and the removal of crowns and bridges. Additionally, older adults (aged 75 or above) can re-apply for another round of dental treatments five years after they have received dental services under this programme [36].

### 5.4. Outreach Dental Care Programme

Older adults receiving long-term care lack self-care ability and often have difficulty obtaining access to conventional dental services. In 2011, the Department of Health launched a three-year pilot scheme and invited NGOs that operated dental clinics to coordinate the services for older adults who lived in residential care homes for the elderly or who received services in day care centres for the elderly [35]. The teams provided basic dental care onsite, such as dental check-ups, scaling, and emergency dental treatments for older adults onsite. They also provided oral care training to caregivers in long-term care facilities and delivered oral health education talks to older adults, as well as their family members and caregivers, in order to enhance their oral health knowledge [23]. In 2014, the pilot scheme was then converted into the Outreach Dental Care Programme for older adults as a regular programme [35]. The scope of treatment has since been expanded to cover restorations, extractions, and dentures [35]. It also expanded to cover more long-term care facilities, such as nursing homes for the elderly who are registered under the Department of Health [35]. Older adults who are in need of further curative therapy can receive treatment onsite or be transported to a dental clinic that the NGO operates [37]. A total of 233,700 older adults received oral health care under this programme from October 2014 to January 2020 delivered by 23 outreach dental teams from 10 NGOs [35].

## 6. Discussion

Hong Kong has a low dentist-to-patient ratio. In 2014, there were 2.13 dentists per 1000 older adults, aged 65 or above, which is far behind many other developed economies [19]. To increase the number of dentists, the government increased the annual number of dental undergraduate places from 53 to 73 (in the 2016–2019 triennium) and further increased to 80 (in the triennium of 2019–2022 triennium). In the same triennium, it also funded 20 dental postgraduate training places annually [38]. Since 2015, the Dental Council of Hong Kong has also increased the number of licensing examinations for non-locally trained dentists to twice a year and has improved the arrangement, in order to attract more qualified dentists to practice in Hong Kong [35].

The Department of Health conducts a community-based oral health survey every 10 years, beginning in 2001, to collect information on oral health status and related behaviour of a few targeted age groups in Hong Kong, in order to facilitate planning and evaluating the oral health programmes [35]. The next round will be conducted in 2021. Additionally, in 2019, the Department of Health set up an expert group, comprising of representatives from the Faculty of Dentistry of the University of Hong Kong (the only dental school in Hong Kong), the Hong Kong Dental Association, the College of Dental Surgeons of Hong Kong, and dental experts from the Department of Health, in order to review and establish oral health goals in different age groups, according to the results from the oral health survey and the local situation [35]. Regarding the surging dental needs for older adults in Hong Kong, as well as the complexity of dental management in older adults, the Department of Health proposed to institute a new dental consultant post for developing and implementing programmes in special care dentistry, such as the Outreach Dental Care Programme for older adults. The consultant would also be responsible for the long-term development of this dental specialty [35].

Some cities in the United Kingdom and France provide public-funded dental services to all people, whereas Hong Kong only provides public-funded dental care for the underprivileged population [39]. However, people with low income in Hong Kong have dental care through the Comprehensive Social Security Assistance Scheme dental grant, which is similar to the Medicare programme that the US federal governments funds [39].

The Hong Kong government has implemented a number of oral health care policies to relieve the increasing dental needs of older adults. All programmes have received substantial support and positive feedback from the public and, hence, have gradually expanded their subsidy amounts, the scope of dental treatments, and the number of eligible older adults gradually over the years. This fulfils the government’s aim to allocate resources to older adults who are in need, such as the Comprehensive Social Security Assistance Scheme, the Community Care Fund Elderly Dental Assistance Programme, and the Elderly Health Care Voucher Scheme for older adults with financial problems, whereas the Outreach Dental Care Programme is for institutionalized older adults with physical difficulties [21]. However, the majority of older adults living in the community (90% of our older adult population) can only benefit from the Elderly Health Care Voucher system, where the annual amount is barely adequate to cover the high dental treatment fees in the private sector. In addition, part of it may be used on other services, such as medical services [19].

Although these programmes are tailored for older adults in need, the utilization rate is relatively low. A 2018 report found that only 14% of older adults had used the elderly health care voucher for dental services within the past year, as they did not perceive any dental need [40]. For the Community Care Fund Elderly Dental Assistance Programme, only 11% of eligible older adults participated as of September 2019. The low participation rate may be due to the fact that the older adults not perceiving any dental need were physically unfit or reluctant to have dental treatment, etc. [33]. To enhance older adults’ utilization of these programmes, we should increase their dental awareness and understanding of the aim of such programmes. Assistance should be provided for scheduling dental appointments. If necessary, transportation or an escort should be provided to remove any physical barriers to accessing these programmes.

In addition, the abovementioned programmes should be regularly evaluated to assess their outcomes and uncover any deficiencies. The most recent evaluation of the Community Care Fund was performed in 2013 and an interim report was generated [32]. However, no formal evaluations have been completed for the Outreach Dental Care Programme and the Elderly Health Care Voucher Scheme for dental services so far. Thus, the government should make an effort to evaluate these programmes’ utilization rates, patients’ satisfaction, feedback from the public and dental service providers, and changes in the oral health status of older adults after they joined the programmes. The evaluations can provide useful information for further improving these programmes.

Due to the limited resources and the high dental treatment costs in Hong Kong, the government mainly provides emergency dental treatment for older adults. The Outreach Dental Care Programme is the only programme that provides preventive measures for institutionalized older adults, in the form of regular dental examinations and the application of topical fluoride. It also promotes oral health, through educational talks and oral care training for older adults and their caregivers. However, the programme is limited to institutionalized older adults, who are a small proportion of older adults in Hong Kong. More oral health education talks should be given to older adults in the community, in order to increase their dental awareness. In the opinion of the authors, more community-based preventive programmes for older adults should be conducted. This is what has been recently emphasized worldwide for the relief of the oral disease burden [41]. The treatment philosophy should change from an expensive intervention-oriented approach to a prevention-oriented approach, in order to tackle oral diseases early on, as well as to address oral health inequalities among people of different sociodemographic backgrounds [41].

With the surging dental need in the growing population of older adults, substantial manpower is needed to provide dental services. The government has implemented several policies to increase the number of dentists who are trained both locally and overseas. However, it is necessary to shift the current dentist-centred model of oral care delivery to a team-approach model, in order to fulfil population-wide oral health needs [40]. Community-based oral care training for non-dentist personnel is needed to ensure a sufficient, well-equipped workforce is available in the future [41].

Aging effects, multimorbidity, and polypharmacy complicate the dental management of older adults [7]. In addition to increasing the number of dental service providers, they should be equipped with knowledge and training in geriatric dentistry. For instance, this subject should be covered more extensively in the undergraduate curriculum. In addition, dental students can be invited to assist in outreach dental services in residential homes and community-based oral health promotion and prevention programmes. This will provide them a better understanding of the dental management of older adults. Geriatric dentistry should also be reinforced for less experienced dentists, through continuing dental education programmes designed to update their knowledge. The subject should furthermore be advocated in postgraduate training with structured courses and clinical training. In fact, a sub-specialty in geriatric dentistry could be set up to help older adults with special needs in the long term.

Dentists and medical doctors should collaborate to maintain both oral health and general health in older adults. Oral health seminars, videos and leaflets should be provided to government outpatient medical clinics for patients with specific medical problems, such as diabetes, cardiovascular disease, and dementia. In 1998, the government established the Elderly Health Service, with 18 elderly health centres currently providing primary health care services for older adults (aged 65 or above) who are living in the community. Their goal is to improve these adults’ self-care ability and to advocate healthy living. However, their services do not include oral health care [42]. It would be more beneficial for these centres to collaborate with dental personnel to provide more comprehensive primary health care for the older adults.

Further, physical barriers remain a major obstacle for older adults seeking access to dental care. A teleconsultation service was started in 1998 in Hong Kong, mainly for older adults living in residential homes, to improve their access to medical care, but the utilization was very limited [43]. During the COVID-19 pandemic, the usage of the teleconsultation service grew in both the public and the private sectors. A 2020 survey found that more than 60% of older adults were willing or very willing to try teleconsultations if the relevant technology was fully developed [44]. Thus, further extending the usage of teleconsultations to dentistry to improve older adults’ access to dental care should be considered.

## 7. Conclusions

Hong Kong, similar to the rest of the world, is facing a growing older adult population with surging dental needs. For this reason, the government has implemented a number of health policies and oral health care programmes. These programmes have received positive feedback and support from the public, as well as from dental service providers. These programmes help older adults maintain adequate oral and general health throughout their lives. There is still room for the further improvement of these programmes in the future.

## Figures and Tables

**Table 1:** The four government-subsidized dental service schemes for older adults in Hong Kong.

Scheme	Eligible Older Adults	Service Providers	Scope of Service
Comprehensive Social Security Assistance (CSSA) Scheme—Dental Grant	Aged 60 or above Have disabilities or are medically certified of poor health	Private dentists Non-governmental organisations	Essential comprehensive dental care, including check-ups, scaling, restoration, root canal therapy, extraction, and removable denture
Elderly Health Care Voucher Scheme	Aged 65 or above Holding Hong Kong Identity Card	Private dentists Non-governmental organisations	Primary dental care
Community Care Fund -Elderly Dental Assistance Programme	Aged 60 or above Non-CSSA recipients Users of home care services or home help service schemes Aged 65 or above receiving Old Age Living Allowance	Private dentists Non-governmental organisations	Essential comprehensive dental care, including check-ups, scaling, restoration, root canal therapy, extraction, and removable denture
Outreach Dental Care Programme	Living in elderly residential care homes or receiving services in day care centres	Outreach dental team Non-governmental organisations	Emergency dental treatment, dental check-ups, scaling, restoration, extraction, and denture Oral care training to caregivers in long-term care facilities Oral health education talks to older adults and their family members or care givers

## Data Availability

Not applicable.
